# Human exploitation of nocturnal felines at Diepkloof Rock Shelter provides further evidence for symbolic behaviours during the Middle Stone Age

**DOI:** 10.1038/s41598-020-63250-x

**Published:** 2020-04-14

**Authors:** Aurore Val, Guillaume Porraz, Pierre-Jean Texier, John W. Fisher, John Parkington

**Affiliations:** 1 Abteilung für Ältere Urgeschichte und Quartärökologie Department, Universität Tübingen, Tübingen, Germany; 2 Evolutionary Studies Institute, Palaeosciences Building, Private Bag 3, WITS 2050 Johannesburg, South Africa; 3 Aix Marseille Université, CNRS, Ministère de la Culture et de la Communication, LAMPEA UMR 7269, FR-13094 Aix-en-Provence, France; 4 Department of Sociology and Anthropology, Montana State University, Montana, USA; 5 Department of Archaeology, University of Cape Town, Cape Town, South Africa

**Keywords:** Archaeology, Social evolution

## Abstract

Within the animal kingdom, carnivores occupied a unique place in prehistoric societies. At times predators or competitors for resources and shelters, anthropogenic traces of their exploitation, often for non-nutritional purposes, permeate the archaeological record. Scarce but spectacular depictions in Palaeolithic art confirm peoples’ fascination with carnivores. In contrast with the European record, research on hominin/carnivore interactions in Africa has primarily revolved around the hunting or scavenging debate amongst early hominins. As such, the available information on the role of carnivores in Anatomically Modern Humans’ economic and cultural systems is limited. Here, we illustrate a particular relationship between humans and carnivores during the MIS5-4 Still Bay and Howiesons Poort techno-complexes at Diepkloof Rock Shelter, South Africa. The recovery of numerous felid remains, including cut-marked phalanges, tarsals and metapodials, constitutes direct evidence for carnivore skinning and, presumably, pelt use in the southern African Middle Stone Age. Carnivore exploitation at the site seems to have focused specifically on nocturnal, solitary and dangerous felines. The lines of evidence presented here suggest the capture and fur use of those felines in the context of highly codified and symbolically loaded cultural traditions.

## Introduction

Interactions with felids are deeply rooted in the evolutionary trajectory of hominins; they manifest themselves in terms of competition, predation and/or exploitation (e.g.^1–7^). In Europe, the identification of carnivore tooth marks on hominin bones points towards a predominantly predator/prey type of relationship between felids and Middle Palaeolithic populations (e.g.^6^). A rare case of Middle Pleistocene hominin exploitation of a large felid (*Panthera leo fossilis*) is documented at the Gran Dolina, Sierra de Atapuerca, in Spain^2^. Coinciding with the cultural bourgeoning associated with the beginning of the Upper Palaeolithic in Europe, these interactions seem to take on a new form, with felids occupying a significant role within symbolic practices of modern human groups. Exploitation of medium (lynx *Lynx lynx*) and large (cave lion *Panthera spelaea*) felids by Upper Palaeolithic people is well documented across Western Europe: canines, either perforated and worn as personal ornaments^8–11^ or used as retouchers^7^, have been recovered from several archaeological assemblages. Felid bones exhibiting cut-marks consistent with skinning and possible fur use are also known from various sites in that region (e.g.^5,11,12^). Felid representations attest to the symbolic value attributed to these predators by Upper Palaeolithic societies. Remarkable examples include the magnificent felid depictions from Chauvet Cave in France^13^ and the therianthropic *Löwenmenschen* ivory figurines recovered from sites in the Swabian Jura^4,14,15^. At La Garma in Spain, the recovery of cut-marked distal phalanges of cave lion *Panthera spelaea* illustrates the use of pelts from this dangerous animal, interpreted in the light of ritual activities during the Magdalenian^5^.

On the African continent, several lines of evidence document predation and competition between medium and large felids and early hominins (see for instance^1,16–18^). There is limited archaeological information, however, on Anatomically Modern Humans’ interactions with carnivores. This contrasts with the ethnographic record, which illustrates a diverse and complex set of interactions between people and carnivores across Africa, generally embedded within highly codified cultural practices. Leopard pelts, for instance, are used to distinguish people from one another, often people of higher status from those of lower status. In Zulu culture in South Africa, wearing a leopard skin is a privilege granted to the king or members of the royal family^19^. In East Africa, amongst Karamojong and Acholi people, the fur of this animal is part of the warrior’s regalia^20^. Lion hunting is instrumental in maintaining the Maasai social structure based on an age-grade system and newly initiated men (*ilmurran*) are expected to know how to hunt lions^21^. Maasai men in Kenya and Tanzania hunt lions for several overlapping motivations, including “achieving and reinforcing the role of *ilmurran* in society” and “to obtain prestige” [21:494].

Symbolically loaded behaviours appear from at least 100,000 BP in Africa, well prior to the advent of the Upper Palaeolithic in Eurasia (e.g.^22–27^). Archaeologically visible proxies for the use of symbols as a means of intra- and inter-human group communication are documented from several Middle Stone Age (MSA) rock shelters in South Africa, often –but not exclusively– in association with the Still Bay and the Howiesons Poort techno-complexes. Still Bay and Howiesons Poort symbolic proxies include the practice of ochre and shell engravings^23,27–30^; the use of perforated marine and terrestrial shell beads as personal ornaments^24,31,32^; and the habitual use of ochre (e.g.^30,33,34^). There is no clear evidence thus far for carnivore exploitation in the context of symbolic practices during the MSA.

Carnivores, although always in very small numbers, are usually present in MSA faunal assemblages retrieved from southern African rock shelters. These assemblages often comprise remains of small (i.e. the African wild cat *Felis silvestris*), medium (i.e. the caracal *Caracal caracal* and/or the serval *Leptailurus serval*) and large (i.e. the cheetah *Acinonyx jubatus* and the leopard *Panthera pardus*) felids. One exception is the largest of all extant felids, the lion *Panthera leo*, never documented from such contexts. Some authors have proposed that the occurrence of carnivores in faunal assemblages could result from skin exploitation by hunter-gatherers. This was suggested for instance at Bushman Rock Shelter^35^ in the interior of southern Africa and at Blombos Cave^36^ in the southern Cape. In one of the Howiesons Poort layers from Klipdrift Shelter, Reynard *et al*.^37^ mention the occurrence of cut-marks on a caracal/serval phalanx, which they interpret as evidence for skinning. In one of the pre-Still Bay layers at Sibudu Cave in KwaZulu-Natal, Clark^38^ describes a series of three parallel cut-marks on the plantar surface of the distal condyle of a small felid (African wild cat size) metapodial, which are consistent with skinning^39^. Evidence for carnivore skin exploitation during the MSA remains, however, tenuous, since it relies solely on this handful of cut-marked bones retrieved from sites spread across the southern African region. Besides, the significance of such practices for human groups is unknown. Here, we present the largest sample of felid specimens yet recovered from a MSA site in southern Africa, with direct evidence of regular skin removal and presumably fur use by humans.

## Diepkloof Rock Shelter, South Africa: Brief Presentation of the site and chrono-cultural-sequence

**Figure 1:**
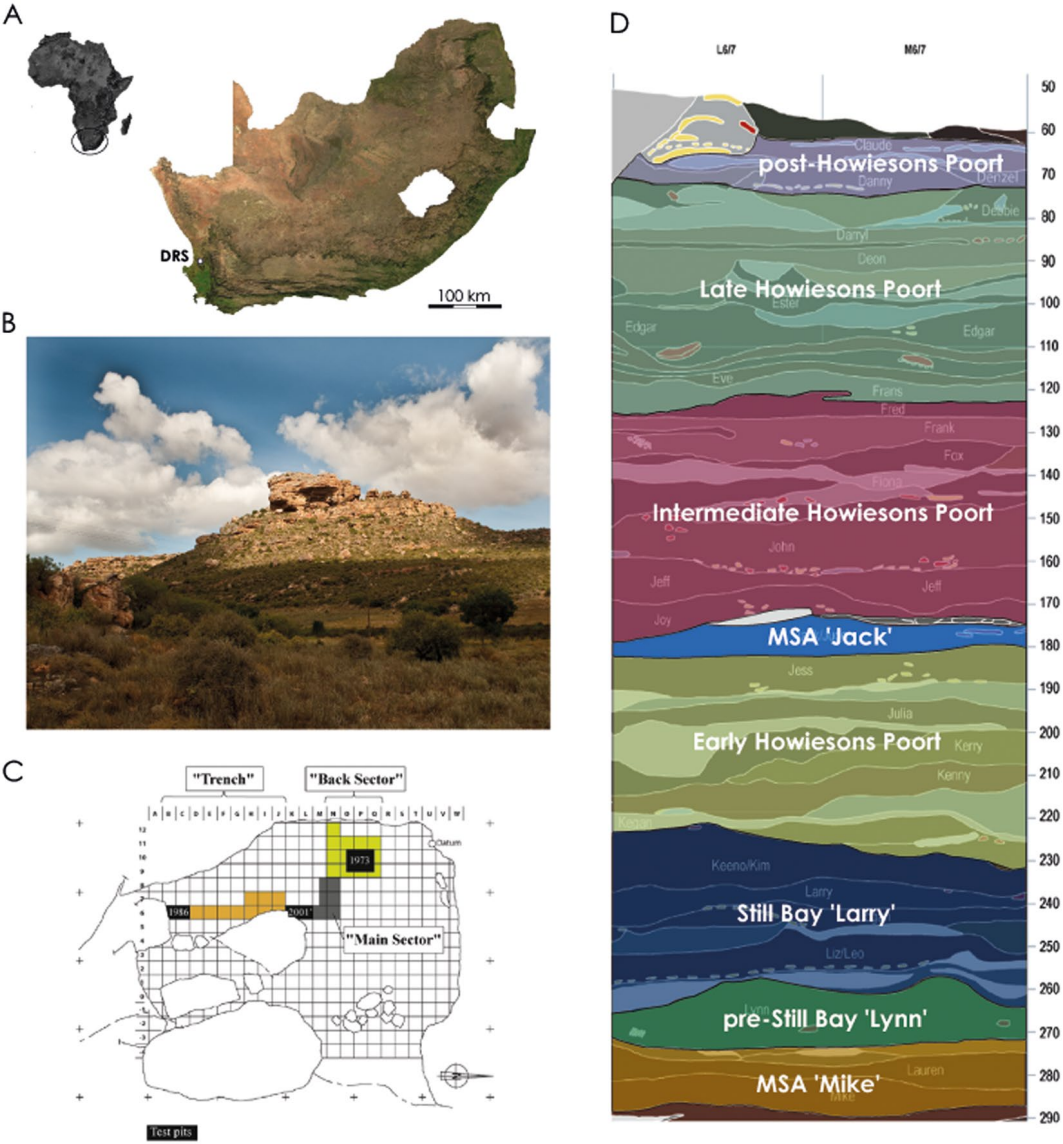
General presentation of Diepkloof Rock Shelter. (**A**) Geographical location in South Africa; (**B**) Northern view of the rock shelter (photography courtesy of C. Hahndiek); (**C**) map of the rock shelter highlighting the different areas of excavation (from^40^); and (**D**) stratigraphic profile in the main sector, in squares L6/L7 and M6/M7 (simplified after^46^).

Diepkloof Rock Shelter (hereafter DRS) is a large and prominent rock shelter located 14 km from the shoreline of the southern Atlantic Ocean in the West Coast of South Africa^40^ (Fig. 1), in the Winter-Rainfall Zone. A perennial river, the Verlorenvlei River, runs about 100 m directly downslope from the rock shelter. Past vegetation, reconstructed using wood charcoal, as well as modern vegetation include a mosaic of plant communities consistent with the open and dry habitats typical of the Fynbos Biome in the Cape Floristic region^41^. These comprise open grassy areas, scrub and shrubs, Fynbos vegetation, as well as patches of Afromontane forest mesic thickets. The proximity of the Verlorenvlei River explains the presence of riverine woodland/wetlands, reed and rush beds^41^.

Excavations at the site from 1999 to 2013 have uncovered a 3.1 m deep archaeological sequence. Deposits are associated with human occupations of the shelter dated from MIS5 to MIS3^42,43^ -but see^44,45^- and consistent with distinct techno-complexes including, for a large part of the sequence, the Still Bay and the Howiesons Poort^46,47^ (Fig. 1). The Howiesons Poort from DRS is unique in its high stratigraphic resolution and chrono-cultural developments, across three successive phases^46^. It is best known for having produced several hundred fragments of engraved ostrich eggs presumably used as containers and marked with geometric motifs^27,48^, a practice shared across a vast territory, from the south of Namibia^49^ to the south coast of South Africa^28^. Regular ochre processing at the site is illustrated by hundreds of shale, ferricrete and shale/ferricrete pieces collected throughout the MSA sequence, including some bearing evidence of grinding, as well as the recovery of tabular quartzite fragments covered in red ochre^33^.

Besides the rich lithic and ochre samples, DRS has yielded a large and taxonomically diverse faunal assemblage, reflecting the various habitats present around the site^50,51^. Existing taphonomic data indicate that Verreaux’s eagle (*Aquila verreauxii*) may be responsible for the accumulation of some of the small mammal, tortoise and bird remains^50,51^. A few coprolites found in one Early Howiesons Poort and two post-Howiesons Poort stratigraphic units suggest visits of the rock shelter by hyenids. While these carnivores might have brought in some of the remains, including the hyenid and canid bones, which are common in hyena-accumulated assemblages, Steele and Klein^50^ identify people as the main accumulators of the faunal assemblage. The diversity of ungulates suggests the adoption of varied acquisition strategies by the inhabitants of the rock shelter, targeting medium to large gregarious individuals as well as smaller solitary ones. Both docile (i.e. the eland *Tragelaphus oryx*) and potentially aggressive (i.e. the extinct long-horned buffalo *Pelorovis antiquus*) large ungulates were hunted^50^. Notwithstanding the impact of anthropogenic overhunting in the last few centuries on carnivore distribution and frequency, the taxonomic composition of the archaeological carnivore assemblage reflects modern distribution of carnivores that still occur in the area^52^. Steele and Klein^50^ mention the presence of nine terrestrial carnivore taxa. They identified the remains of canids (the Cape fox *Vulpes chacma* and the black-backed jackal *Canis mesomelas*), several small carnivores (the honey badger *Mellivora capensis*, the Cape grey mongoose *Galerella pulverulenta* and a genet *Genetta* sp.), a hyenid (Hyaenidae gen. et sp. indet.), and felids, namely *Felis silvestris libyca*, *Caracal caracal* and/or *Leptailurus serval*, and *Panthera pardus*. Felids overwhelmingly dominate the carnivore sample, with more than 75% of the carnivore remains (NISP^50^).

## Sample studied and results

### Stratigraphic origin of the remains

In this study, we consider only felid remains (n = 61). Most of these (n = 40) come from the stratigraphic deposits that have been exposed from the “Main Sector”, while the others come from the “Trench” (n = 13) and from the “Back Sector” (n = 8)^40^ (Fig. 1; Table 1). The majority of the felid remains come from stratigraphic units assigned to the Still Bay ‘Larry’, the three Howiesons Poort phases and the MSA ‘Jack’ (Table 1). One bone comes from the MSA ‘Mike’ and another one from a post-Howiesons Poort layer. No felid remains were identified in the pre-Still Bay ‘Lynn’, a techno-complex represented by one stratigraphic unit only^46^.

**Table 1:** Stratigraphic provenience of the felid remains per techno-complex in the Diepkloof Rock Shelter archaeosequence (data provided respectively in NISP and MNI; SB: Still Bay; HP: Howiesons Poort).

Species	MSA Mike	Pre-SB Lynn	SB Larry	Early HP	MSA Jack	Interm. HP	Late HP	Post-HP
*Panthera pardus*	—	—	—	1/1	1/1	1/1	—	—
*Caracal caracal*	—	—	2/1	—	—	3/1	9/1	—
Caracal/serval	1/1	—	4/1	1/1	—	2/1	9/1	—
*Felis silvestris*	—	—	7/1	3/1	3/1	5/1	8/2	1/1
TOTAL	1/1	—	13/3	5/3	4/2	11/4	26/4	1/1

### Taxonomic composition of the felid assemblage

The felid remains belong to a minimum number of 18 individuals (Table 1). Three specimens are attributed to the leopard and were recovered from distinct techno-complexes, thus representing a minimum number of three individuals. Twenty-seven remains belong to the African wild cat and represent a minimum number of seven individuals. Half of the remains in the felid sample (n = 31) belong to either the caracal or the serval. Modern *C. caracal* and *Leptailurus serval* present similar cranial and post-cranial morphological features and both species are characterized by significant sexual dimorphism. While the larger remains (n = 14) could confidently be assigned to a minimum number of three adult males *C. caracal*, others (n = 17) fit the dimensions of either smaller female caracals or larger male servals. These remains are therefore included in the “caracal/serval” category and represent a combined minimum number of five individuals. A consideration of modern distribution of these two taxa indicates that servals are absent from the Fynbos Biome^52^. Today, servals occur only in the eastern part of KwaZulu-Natal and in Limpopo, in the Summer-Rainfall Zone. They require proximity to water and adequate cover. Caracals are well documented in the Winter-Rainfall Zone, including along the West Coast; they inhabit open savannah woodland and thrive particularly in open vleis^52^, a type of habitat present today and documented in the past around DRS^41^. Although we have cautiously decided to maintain a “caracal/serval” category due to possible alterations of the serval distribution map between the MIS5-MIS3 and today, we do suggest that most of the remains from this category are more likely to belong to caracals than to servals.

### Skeletal element representation

**Figure 2:**
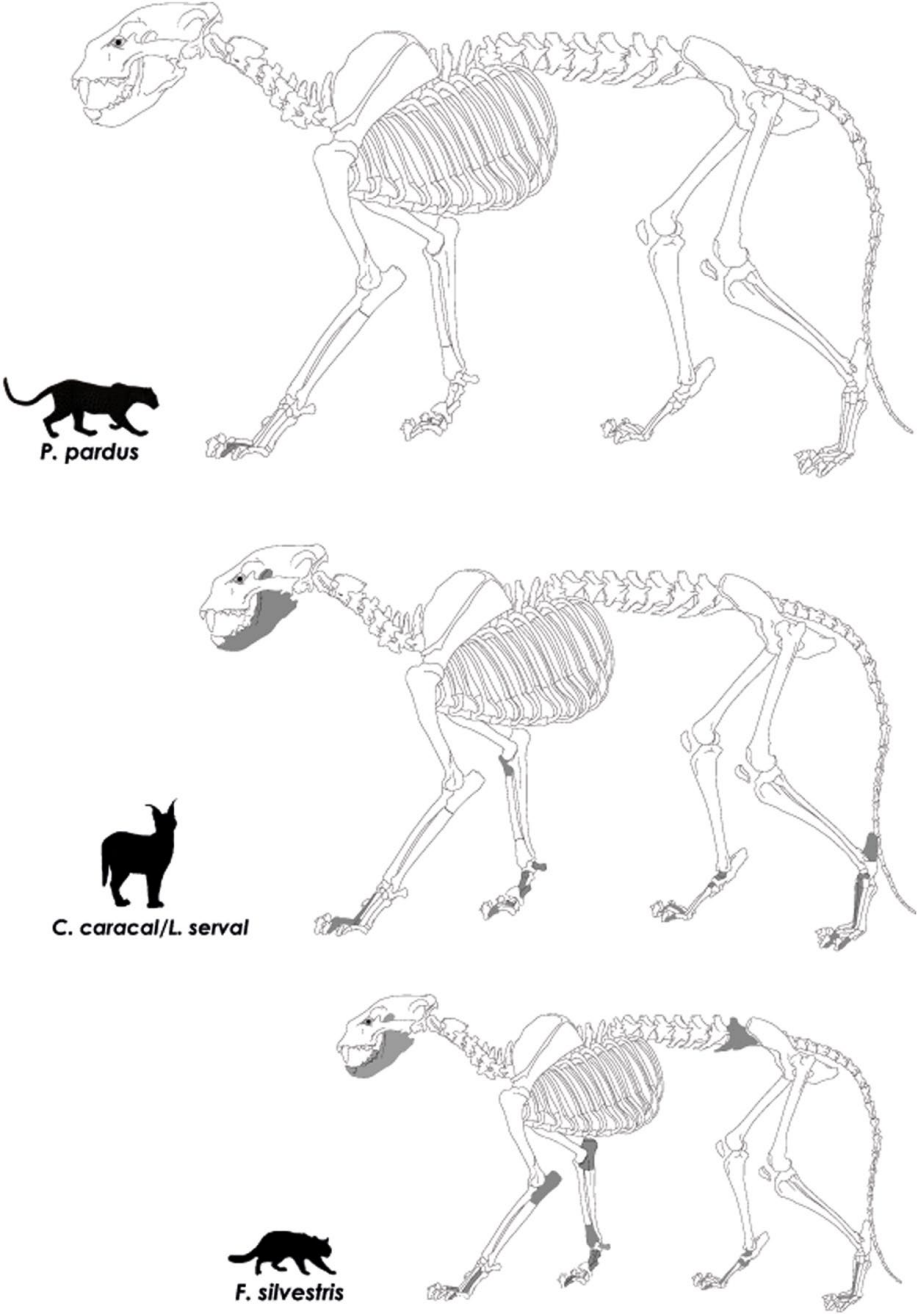
Skeletal elements preserved for leopard, caracal/serval and African wild cat.

Most of the felid skeletal parts preserved (50/61 or 82%) are consistent with bones from the autopod: more than a third of the assemblage comprises proximal and intermediate phalanges (claws are absent), a quarter carpals/tarsals and a fifth metapodials, while other skeletal elements represent less than a fifth (Table 2; Fig. 2). There is no diachronic pattern regarding the stratigraphic provenience of autopod versus other skeletal parts, all evenly distributed across the archaeological sequence. The leopard remains include the distal half of a proximal phalanx, one complete and one fragmentary intermediate phalanges. The caracal/serval remains include five fragmentary proximal phalanges, three fragmentary and six complete hind and front intermediate phalanges, one complete pisiform, one proximal calcaneum, one fragmentary and one complete talus, two complete naviculars, one complete cuboid and one complete medial cuneiform. Other skeletal parts preserved are a partial mandible with dentition and two proximal radii. The African wild cat sample comprises one complete and two fragmentary hind proximal phalanges, five complete intermediate phalanges, a complete pisiform, two complete cuboids, a complete talus and a complete navicular, a complete first metacarpal, one fragmentary third and fourth metacarpals and a fragmentary second metatarsal. Besides elements from the autopod, the African wild cat sample also includes two partial mandibles with dentition, three proximal ulnae, two lumbar vertebrae and one distal radius.

**Table 2:** Anatomical distribution of the felid skeletal elements (data in NISP and %NISP).

Species	Total NISP	Phalanges	Carpals/Tarsals	Metapodials	Others
Leopard	3	3	0	0	0
Caracal	14	7	2	3	2
Caracal/serval	17	7	6	3	1
African wild cat	27	8	5	6	8
TOTAL	61	25/41	13/21.3	12/19.7	11/18

### Human-induced bone surface modifications

Forty-four felid remains exhibit different stages of discoloration, from dark brown to white (calcined), resulting from burning (Table 3). Seventeen remains bear microscopic trampling striations on their surface (Table 3). Three caracal/serval long bones preserve evidence of green breakage: both parts of a broken metacarpal shaft; the proximal edge of a broken distal second metatarsal; and the distal edge of a broken proximal third metacarpal. A distal radius of a juvenile African wild cat also presents the characteristic of a bone that was broken while still fresh.

**Table 3:** Human-induced bone damage and surface modifications (data in NISP and %NISP).

Species	Skeletal element	Burning	Trampling	Green breakage	Cut-marks
Leopard	Phalanges	3	1	0	2
Caracal + caracal/serval	Phalanges	12	5	0	5
	Metapodials	2	1	3	1
	Carpals/Tarsals	4	1	0	3
	Long bones	1	1	0	2
African wild cat	Phalanges	7	2	0	0
	Metapodials	4	3	0	1
	Carpals/tarsals	4	0	0	1
	Long bones	4	2	1	1
	Vertebra	2	1	0	0
	Mandible	1	1	0	0
TOTAL		44/72.1	17/27.9	4/6.6	16/26.2

**Figure 3:**
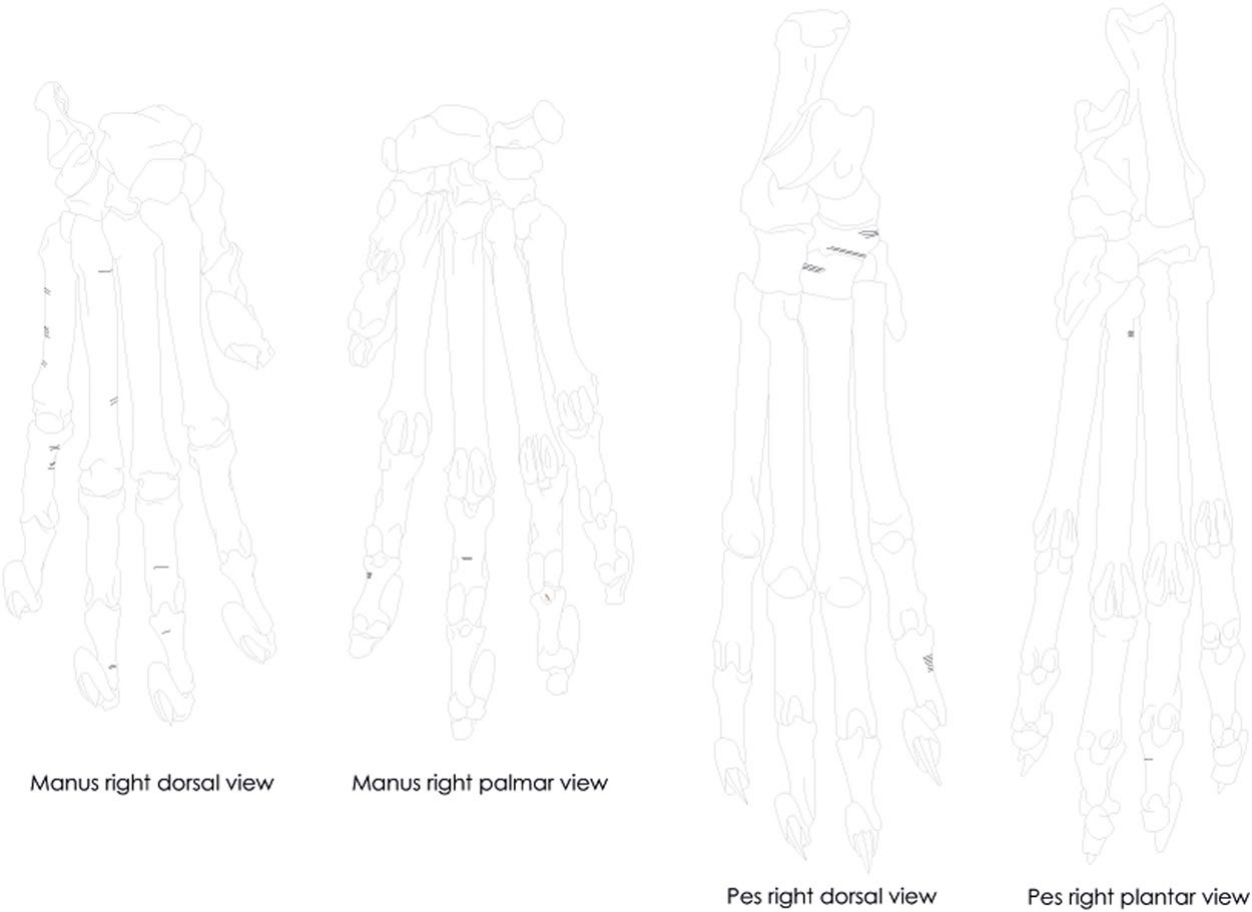
Cut-marks observed on felid phalanges, carpals, tarsals and metapodials, here combined on right felid manus and pes.

**Figure 4:**
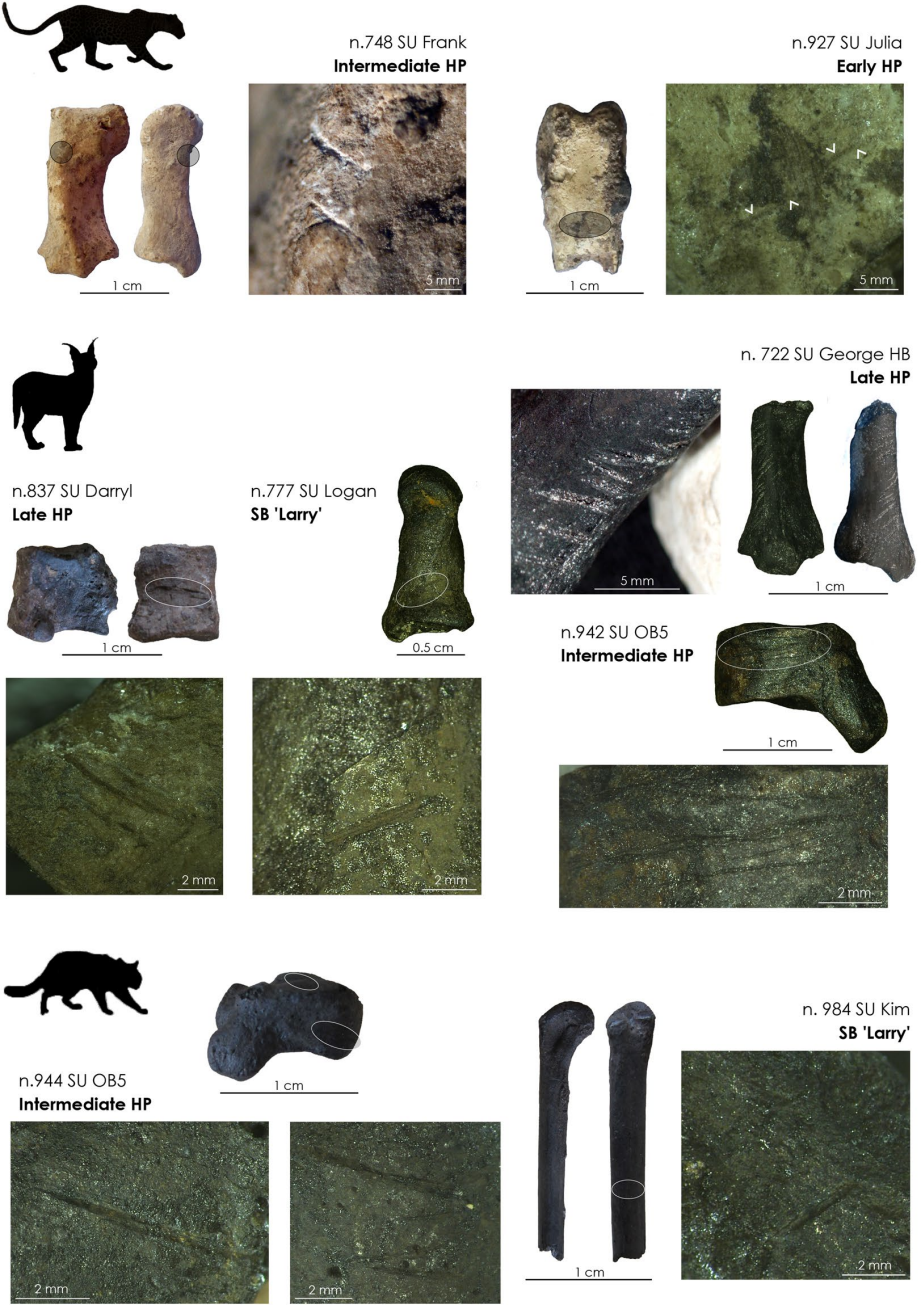
Examples of leopard, caracal/serval and African wild cat cut-marked metatarsals, tarsals and phalanges retrieved from Still Bay (SB) and Howiesons Poort (HP) stratigraphic units.

Sixteen remains, mostly elements from the autopod (n = 13), exhibit butchery marks produced by cutting with stone artefacts (Table 3; Figs. 3 and 4). These remains include two of the three leopard phalanges; a distal radius, a talus, and a fourth metatarsal of African wild cats; a proximal radius, a third metatarsal, and two intermediate phalanges of caracals; and a proximal radius, a navicular, a talus, a medial cuneiform, one proximal and two intermediate phalanges of caracals/servals. Figure 3 combines the location and orientation of cut-marks observed on the felid autopod bones. On phalanges, metapodials and tarsals, the cut-marks are short, transverse and located on both the dorsal and palmar/plantar sides (Figs. 3 and 4). On the caracal/serval proximal radii, the cut-marks are transverse and located on the anterior face of the shafts. We did not observe cut-marks on the cranial material or on the vertebrae. With the exception of one caracal/serval phalanx from stratigraphic unit ‘Lauren’ in the MSA ‘Mike’, all cut-marked bones come from Still Bay ‘Larry’ (n = 4), Early (n = 1), Intermediate (n = 5) and Late (n = 5) Howiesons Poort stratigraphic units.

The African wild cat distal radius broken while fresh also exhibits a cut-mark. Eleven of the sixteen cut-marked specimens are burnt and trampling marks are present on six of the cut-marked bones.

## Discussion and conclusion

### Exploitation of felines for their fur at Diepkloof Rock Shelter

While not documented for felids, there is available information on small (*Vulpes vulpes* and *V. velox*) and large (*Canis familiaris*, *C. lupus* and *C. latrans*) canid bone density^53^. Canids and felids are cursorial quadrupeds, presenting similar skeletal architecture and dimensions and we therefore used published observations on canid bone mineral volume density as a comparative sample to investigate whether the DRS felid assemblage has suffered from density-mediated attrition. Canid skeletal elements with the highest density are the middle shafts of long bones (femur, tibia, fibula, and humerus), mandible, calcaneum, and middle shaft of metapodials; elements with the lowest density are the proximal and distal parts of ribs, centrum of thoracic vertebrae, proximal humerus, proximal tibia, sacrum and greater trochanter of the femur^53^. At DRS, the felid elements preserved are all consistent with some of the densest skeletal parts in canids. Carpals and tarsals, overrepresented at DRS, are also compact bones, which are less prone to fragmentation and easier to identify than long and flat bones. The absence of most felid long bones could partly be due to high fragmentation leading to their non-identification, especially since identifiable portions in long bones are epiphyses, which present some of the lowest densities in canids^53^.

Density-mediated attrition therefore seems to have strongly influenced the DRS felid skeletal element representation. The felid remains have also suffered from human-induced alterations in a similar manner to the ungulate assemblage (i.e. burning, trampling and intense fragmentation^50^). This confirms that the occurrence of these remains amongst the archaeological deposits likely results from anthropogenic processes. The anthropogenic origin of the felid bones is further supported by the fact that caracals/servals and African wild cats are not particularly prone to using rock shelters and caves^52^. Leopards on the contrary are cave-dwelling, bone-accumulators carnivores (e.g.^1,54^). At DRS however, there is only limited evidence for carnivore contribution to the faunal assemblage and this evidence points towards a contribution by hyaenids rather than by felids^50^. Finally, the percentage of cut-marked bones within the felid assemblage is high, even when compared to the ungulate assemblage, primarily accumulated by the inhabitants of the rock shelter, which comprises only seven remains with butchery marks^50^.

The skeletal element representation observed for the felid assemblage at DRS is reminiscent of the head and foot-dominated pattern regularly observed for ungulate assemblages from archaeological sites and resulting from the combination of taphonomic variables including density-mediated attrition as well as biases related to excavation and analytical procedures (see^55^ and references therein). The survival pattern observed for felid remains at DRS is neither completely consistent with a typical pelt processing scenario nor with a scenario where used skins would have been discarded. In the first case, most skeletal parts should be preserved with the exception of elements left in the furs (phalanges, caudal vertebrae and possibly skulls), while the inverse skeletal part representation would be observed in the second case^11^. At DRS, the scenario is, unsurprisingly, more intricate and probably combines both skinning and pelt discarding events. This is to be expected given the relatively small sample sizes per techno-complexes, the fact that the remains come from a sequence that covers a long chronological interval, the various taphonomic agents that have affected the assemblage, and the small size of the excavation area in comparison with the total surface of the rock shelter (Fig. 1).

The location and orientation of cut-marks observed on the DRS felid bones are all consistent with skinning motions. Similar transverse cut-marks located on tarsals, carpals, metapodials, phalanges and the anterior face of the radius were produced during experimental skinning of small to medium carnivores^11,39,56,57^. Skinning cut-marks occur on parts of the skeleton not covered by muscles, where direct contacts between the implement cutting through the skin and the bone surface can occur. At DRS, we did not observe cut-marks on any of the three fragmentary felid mandibles. This contrasts with experimental skinning, which tends to produce abundant cut-marks on the ramus and condyle of the mandible^11,39,56,57^, unless of course the skull remains inside the fur. Skeletal elements most likely to bear cut-marks related to butchery motions other than skinning -namely disarticulation and defleshing- are absent from the assemblage. From existing observations, there is no evidence for meat removal. Interestingly, claws, which are relatively easy to identify and characterized by moderate mineral volume densities in canids^53^, are absent from the assemblage. They could have been extracted, used and discarded outside of the site. Alternatively, their absence at the site, combined with the placement of cut-marks and considering that skinning marks cannot occur on elements left inside the fur^11,39,56^, could suggest that skinning at DRS was performed by carefully removing the fur until the lowest possible level and cutting it between the intermediate phalanges and the claws, which would have been left inside. Other skinning methods would involve cutting the fur higher up (i.e. at the wrist/ankle joint or between the metapodials and the proximal phalanges^11,56^) and would result in more bones -not bearing cut-marks- left inside the fur. The pattern observed at DRS speaks in favour of the intentionally careful removal of the most complete possible furs, presumably in anticipation of their use.

### The Diepkloof Rock Shelter felids in the context of carnivore exploitation by late Pleistocene populations in South Africa

Although always in small numbers, when felids are present in MSA assemblages they tend to be more abundant than hyenids and canids combined^36–38,58–64^. Table 4 compares the DRS felid sample with observations from other South African MSA assemblages, which have also yielded felid remains. Both the leopard and the African wild cat have been identified from the MSA deposits at Klasies River Mouth and Border Cave but published information on these assemblages^58,59^ is provided in MNIs and is therefore not included in this table.

**Table 4:** Felid remains recovered from other MSA sites in southern Africa. The observations, provided in NISP and % of the total NISP for the mammalian assemblages, come from: ^63^ for Ysterfontein (YST); ^37^ for Klipdrift Shelter (KDS); ^36,60,61^ for Blombos Cave (BBC); ^62^ and^38^ for Sibudu Cave (SIB); and ^64^ for Bushman Rock Shelter (BRS).

	DRS	YST	KDS	BBC	SIB	BRS
African wild cat	**27**	7	—	23	—	—
Small felid	—	—	—	1	7	—
Caracal/serval	**31**	13	2	3	—	1
Medium felid	—	—	—	—	1	1
Leopard	**3**	—	—	—	—	—
Cheetah	—	—	—	—	1	—
Large felid	—	—	—	—	4	2
Lion	—	—	—	—	—	—
Total	**61**	**20**	**2**	**26**	**13**	**4**
NISP mammalian fauna	4173	3324	2266	6529	6907	1822
%NISP	1.5%	0.6%	0.1%	0.4%	0.2%	0.2%

When considering both raw values (NISP) and percentages of the complete mammalian assemblages represented by felids (%NISP), the largest sample comes from DRS (Table 4). While still only representing a very small fraction of the fauna, the larger size of the DRS felid sample compared to other MSA sites is statistically significant (all Fisher’s Exact tests produce p values < 0.0005). We exclude inter-specialist variability as a possible bias having affected taxonomic identifications. The assemblages were indeed analysed by a limited number of specialists (and by the same one in several cases:^50,58–60,63^), using similar methods and in many cases the same comparative collections (i.e. from the Ditsong Natural History Museum in Pretoria:^36–38,61,62,64^; this study). We also exclude differential preservation between faunal assemblages as a significant bias since the MSA fauna from DRS is characterized by intense fragmentation and poor bone preservation^50^. There is no information published on felid skeletal part representation for other MSA assemblages, which hinders comparisons with DRS at this stage.

The recovery of felid bones exhibiting skinning cut-marks from the MSA ‘Mike’ at DRS and the pre-Still Bay at Sibudu Cave^38^ indicate that human exploitation of these carnivores is not restricted to the Still Bay and the Howiesons Poort, although evidence from DRS suggests a stronger signal for such behaviours during these two techno-complexes. At DRS, felid remains disappear in the post-Howiesons Poort stratigraphic units, pointing towards the abandonment of this practice. The one felid bone with skinning marks from Klipdrift Shelter comes from one of the Howiesons Poort stratiraphic units^37^, while the second largest concentration of felid material (17 African wild cat remains) noted for an MSA assemblage after DRS is from the phase M1 at Blombos Cave, attributed to the Still Bay^36,60^. Several leopard, caracal/serval and African wild cat remains were also retrieved from the MSA II of Klasies River Mouth (MNIs, respectively, of nine, three and one^58^). Hyenid bones are extremely scarce in MSA faunal assemblages and no cut-marked specimen is reported in the literature. For canids, there is only one mention of cut-marks on a jackal ulna from an Intermediate Howiesons Poort stratigraphic unit at DRS^50^.

### Behavioural implications of capturing nocturnal, solitary and dangerous felines

The three feline taxa identified at DRS share several ethological traits that make them difficult to acquire, dangerous prey. Leopards, caracals and African wild cats are primarily nocturnal and solitary and, as secretive predators hunting by stalking their prey, they have developed particularly good talents at making themselves extremely elusive and therefore difficult to spot^52^. There are documented modern cases of humans being eaten by leopards (e.g.^1,65,66^) and a leopard under threat, for instance trapped, would be extremely aggressive^1,52^. While caracals and African wild cats are not powerful enough to represent life threats to humans, encounters with these predators could still result in serious wounds for the hunters.

These observations, combined with archaeological evidence for skinning of felines during the MSA at DRS, suggest that the relationship between human and felines, and specifically the human interest in these carnivore pelts, was embedded in a wider development amongst late Pleistocene people’s involvement with marking – of objects, of places and of people. Previous reporting of artefactual material from DRS and other pene-contemporary occupations for instance at Sibudu Cave, Blombos Cave and Klipdrift Shelter illustrates the regular involvement of MSA hunter-gatherers, especially during the Still Bay and Howiesons Poort, in marking of themselves, their possessions and their surroundings. The geometric markings on ostrich eggshells from DRS^27,48^ and Klipdrift Shelter^28^ transform generic eggshells into specific, distinguishable objects that carry recognisable meanings, likely marks of ownership. Perforated marine shell beads retrieved from Still Bay contexts at Blombos Cave and Sibudu Cave^24,31,34^ are items of personal ornaments worn in the context of symbolically loaded social interactions. The spatial organization of shelter occupations and the repetitive depositional arrangements of bedding and hearth areas at DRS^67^ and Sibudu Cave^68^ transform a location into a place, likely a home claimed and destined for re-use. The increased use of ochre and the deployment of decorative beads strung together transforms a man or a woman into a particular person, with particular relationships and allegiances. These are all markings that turn the generic into the specific and begin to demarcate meanings and establish visible claims on property, places and identities. We suggest that it is in this sphere of marking, of distinguishing, of identifying, that the interest in skinning dangerous carnivores at DRS should be set.

## Methods summary

The felid remains presented here were selected from the MSA faunal material of DRS, which is currently curated by the Department of Archaeology at the University of Cape Town (UCT), South Africa. We produced initial anatomical and taxonomical attributions using the modern reference collections from the UCT department and completed them with the more extensive one from the Ditsong National Museum of Natural History in Pretoria. Specimen numbers provided in this paper follow our own, independent numbering system. For the minimum number of individual (MNI) estimates, we followed the definition proposed by Klein and Cruz-Uribe^69^ that takes into account ontogenetic age information when available (juvenile or adult based on a simple description of the fusion degree for long bones, i.e. unfused or fused). For the MNI counts, felid remains retrieved from different stratigraphic units within a given techno-complex were considered together and we have interpreted the felid remains from different techno-complexes as belonging to different individuals. For instance, seven African wild cat remains were collected from four distinct stratigraphic units attributed to the Still Bay ‘Larry’. They have been pulled together, resulting in a MNI of one rather than four. The samples were always too small (NISP = > 10 per taxon and per techno-complex) to provide statistically meaningful %MAU counts.

We conducted a systematic microscopic investigation of bone surface modifications using an Olympus SZ61 optical microscope offering magnifications up to x45, under oblique lighting. We firstly estimated the visibility degree of bone surfaces (0–25, 25–50, 50–75, >75%), before attributing a degree of manganese coating following Val^70^. We recorded the presence/absence of the following abiotic and biotic modifications: water abrasion, dissolution, trampling, root and rootlet etching, decalcification, crystals, concretions, gastric acid etching, rodent gnawing, invertebrate damage, carnivore damage (pits, punctures, scores, furrows and crenulated edges), bird of prey damage (scoring, notches, beak/talon impacts), and anthropogenic modifications (cut-marks, percussion marks, and polishing). The identification of the biotic and abiotic damage was based on the personal experience of one of us (AV) and on available literature on these aspects, notably:^1,71–75^. A colour-code tentatively linked to a burning stage (cream/unburnt; dark brown/slightly burnt; black/carbonized; grey; white/calcined) following Stiner *et al*.^76^ and Reynard *et al*.^37^ was applied. The description of the long bone breakage patterns uses criteria proposed by Villa and Mahieu^77^ to distinguish between green and dry-breakage. Cut-marks were reported on Inskape templates. Their interpretation in terms of specific butchery motion (i.e. skinning, defleshing, or disarticulation) and skinning method(s) is based primarily on butchery marks produced experimentally by taxidermists on 19 small and medium carnivore carcasses and described in Val & Mallye^39^. Other works describing cut-mark patterns produced during experimental butchery of small carnivores were also consulted^56,57^. Quantitative comparisons between the percentage of felid bones compared to whole mammalian assemblages at DRS and other MSA sites were performed using Fisher’s Exact tests to account for the small sizes of some of the samples (calculations were done using the PAST 3.x software).

## Data Availability

The dataset generated during the current study is available from the corresponding author on reasonable request.
